# Associations between adhering to 24-hour movement guidelines and anxiety among adolescents across intersectional identities: KYRBS 2020–2022

**DOI:** 10.1186/s12889-025-24215-9

**Published:** 2025-09-24

**Authors:** Suryeon Ryu, Eun Young Lee, Zan Gao

**Affiliations:** 1 Center for Children’s Healthy Lifestyles & Nutrition, Children’s Mercy, Kansas City, MO United States of America; 2 School of Kinesiology & Health Studies, Queen’s University, Kingston, ON Canada; 3 Department of Sport Industry Studies, Yonsei University, Seoul, South Korea; 4 Department of Kinesiology, Recreation, and Sport Studies, University of Tennessee, Knoxville, TN United States of America

**Keywords:** Mental disorders, Preventive medicine, Physical activity, Sedentary behavior, Sleep

## Abstract

**Background:**

Identifying preventive measures for adolescent mental health is a global public health priority, as poor mental health heightens the risk of early death from related issues, including suicide. This study examined the associations between adherence to the 24-Hour Movement Guidelines and anxiety among a nationally representative sample of South Korean adolescents, using an intersectional identity framework.

**Methods:**

Data from the 2020–2022 Korea Youth Risk Behavior Surveys were analyzed. Adolescents aged 12–17 years were grouped based on adherence to guidelines: none, physical activity (PA), sedentary behavior (SB), sleep, two guideline combinations, and all three. Anxiety was the mental health outcome. Logistic regression analyses were conducted by intersectional identity groups, adjusting for age, academic performance, family economic status, and body mass index.

**Results:**

Among 122,284 adolescents (48.3% female, mean age 14.8 ± 1.5 years), anxiety prevalence was higher in females. Sleep adherence was associated with lower odds of anxiety for both sexes, with stronger associations observed in males (OR: 0.50, 95% CI: 0.38–0.66) and females with high academic performance on weekdays (OR: 0.48, 95% CI: 0.36–0.64). Stronger associations were seen in females from high-income families (OR: 0.48, 95% CI: 0.37–0.63). While SB adherence alone was not linked to lower anxiety, combined adherence to sleep and SB was associated with lower odds of anxiety. PA adherence was associated with increased odds of anxiety, particularly among females across academic and economic groups.

**Discussion:**

Adherence to sleep guidelines was linked to reduced anxiety, highlighting the crucial role of sleep duration in anxiety management. This underscores the need to consider adolescents’ diverse backgrounds when recommending movement behaviors, warranting further investigation in implementation studies.

**Supplementary Information:**

The online version contains supplementary material available at 10.1186/s12889-025-24215-9.

## Background

Mental health is integral to overall health and well-being, yet reports of anxiety, increased globally by 25.6% during the COVID-19 pandemic [1]. The pandemic’s disruptions, such as social isolation, interrupted education, and economic instability, exacerbated pre-existing mental health disparities, with higher prevalence observed among females, younger people, and low- and middle-income communities [1]. Academic performance, closely tied to career prospects, significantly influences adolescent mental health, particularly in academic-achievement oriented societies [2]. The onset of mental health conditions is often first observed during adolescence, potentially leading to persistent issues into adulthood if left untreated [3]. This is a concern, as poor mental health increases the risk of premature death from interconnected health problems, including suicide [4].

Suicide is the second leading cause of death among young populations, and reducing adolescent suicide attempts is a key objective of the Healthy People 2030 initiative [5, 6]. In the United States, the rate of suicide attempts among adolescents increased to 10.2 per 100 in 2021, reflecting a 38% rise from 7.4 in 2017 [6]. Similarly, in South Korea (hereafter Korea), youth suicide mortality continues to rise, reaching 11.0 per 100,000 individuals in 2021 compared to 7.2 in 2017 (53% increase) [7]. As a result, identifying potential preventive measures for mental health conditions during adolescence is a critical global public health priority.

Adopting a healthy 24-hour lifestyle, including regular participation in moderate-to-vigorous physical activity (MVPA), reduced sedentary behavior (SB), and sufficient sleep, is a key preventive approach to overall health and well-being among adolescents [8, 9]. The Canadian 24-Hour Movement Guidelines provide an evidence-based foundation for comprehensive health promotion interventions [10]. The Guidelines recommend that adolescents should engage in at least 60 min of daily MVPA and limit leisure screen time to 2 h per day, and get 9 to 11 h of sleep for those aged 5 to 13 years, and 8 to 10 h for those aged 14 to 17 years [8]. However, the COVID-19 pandemic significantly disrupted these movement behaviors among Korean adolescents, with lockdowns, school closures, and public health restrictions leading to adverse lifestyle movement patterns [11]. Understanding how these interdependent movement behaviors are associated with mental health conditions, especially in combination, can offer meaningful insights for developing cost-effective preventative strategies to promote adolescents’ mental well-being [8]. Despite the research highlighting the positive effects of healthy movement behavior profiles, disparities in these behaviors remain largely unaddressed. There is still a lack of comprehensive evidence on the relationship among 24-hour movement behaviors and mental health conditions, particularly regarding how these associations vary among different intersectional identity groups. Further investigation is needed to uncover the heterogeneity in these behaviors and their mental health outcomes among diverse populations [12]. 

As known, individuals belong to multiple intersectional identity categories, making it essential to employ an intersectional framework to better capture the intricate individual and societal factors contributing to health disparities in adolescent populations [12]. The concept of intersectionality, introduced by Crenshaw [13], underscores that health outcomes are shaped by the interactions among multiple identities, not by any single identity alone [14, 15]. When analyzing health disparities and inequalities, various social inequities intersect and amplify one another, resulting in a range of unequal health outcomes at both the individual and population levels within systems of oppression [14, 15]. This perspective enables a more accurate identification of the root causes of health disparities and ensures that research findings are relevant to real-world contexts where these intersecting forms of oppression exist [14, 15]. 

While extensive research has been conducted in Western contexts [16], there is a significant lack of evidence from East Asia, particularly Korea. One study explored six-year trends and intersectional correlates of meeting the 24-Hour Movement Guidelines among Korean adolescents, utilizing repeated cross-sectional, nationally representative data through an intersectional lens [17]. The findings revealed that adherence to individual and overall 24-Hour Movement Guidelines was generally low (< 1% for meeting all recommendations) and was notably influenced by gender. The study also emphasized that applying intersectionality in behavioral research is a useful statistical framework in identifying important correlates of movement behaviors and health. Given the poor behavioral profiles of Korean adolescents, coupled with rising mental health concerns and increasing suicide rates, which have been further exacerbated by the COVID-19 pandemic, it is both timely and essential to examine the relationship between movement behaviors and anxiety among Korean adolescents.

This study investigated the associations between adherence to the 24-Hour Movement Guidelines and anxiety, a prevalent mental health issue among adolescents, using representative Korean data from 2020 to 2022. The analysis focused on different intersectional identity groups, hypothesizing that meeting each guideline would be associated with lower anxiety, and adhering to two or more recommendations would further reduce anxiety, with variations across different intersectional identity groups. The findings of this study can reveal unique patterns of behavioral profiles and mental health conditions among adolescents in Korea, particularly during the COVID-19 pandemic, and thereby assisting researchers, educators, and health professionals to deepen their understanding of globally relevant insights and non-pharmacological strategies to support adolescents with diverse identities increasingly susceptible to anxiety. By applying an intersectional approach, this study aims to identify effective strategies to enhance health equity, especially for vulnerable adolescents who face heightened mental health challenges.

## Methods

### Research design and data source

This study utilized de-identified, publicly available, repeated cross-sectional data from the Korea Youth Risk Behavior Web-Based Survey (KYRBS) collected among a representative sample of Korean adolescents in 2020, 2021, and 2022 [18–20]. The KYRBS, conducted annually by the Korea Centers for Disease Control and Prevention (CDC) in collaboration with the Ministry of Education and the Ministry of Health and Welfare, has monitored adolescent health-related measures to inform government policies [21]. Data collection occurred from August to October of each year, with trained teachers explaining the survey’s purpose and procedures to students. In schools where administering the survey in computer education classes was not possible due to the COVID-19 pandemic, students completed the survey using mobile devices (e.g., mobile tablets or smartphones) in their home classrooms.

### Sample

**Fig. 1:**
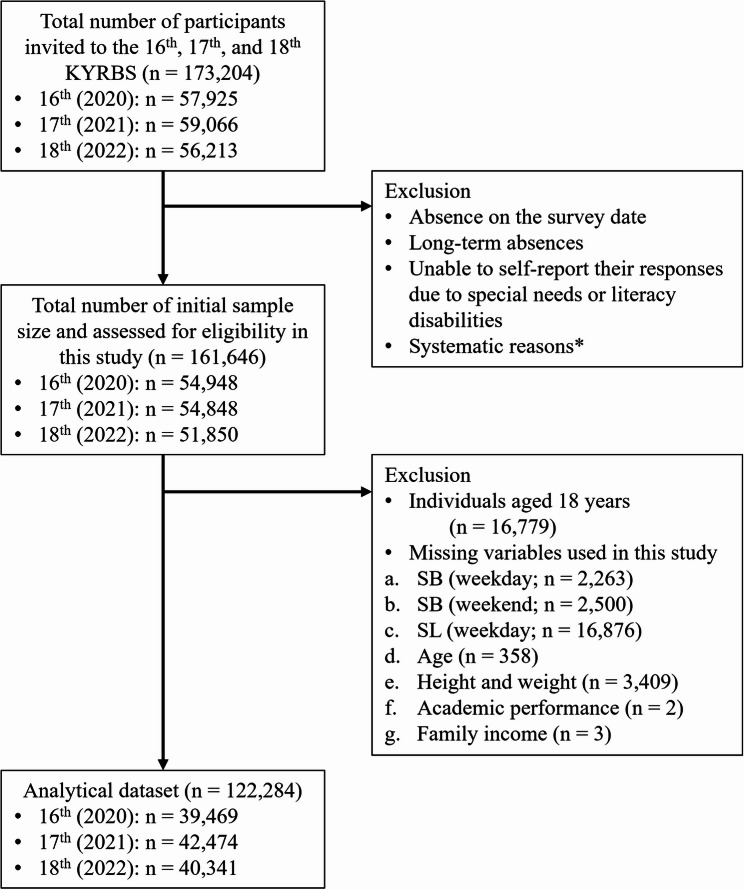
Flow Chart

The target population included middle and high school students at the time of data collection nationwide. A complex, multi-stage sampling method was used to randomly select 400 middle schools and 400 high schools, with one classroom from each grade level within each school chosen randomly. All students present in the selected classrooms on the day of the survey were invited to participate. Eligible participants included students who attended school regularly, without long-term absences, and were able to complete a self-reported online survey. Students with prolonged absences, special needs requiring assistance, or literacy challenges were excluded from the sample. For this study, data from three separate datasets were combined. From the initial total of 161,646 students, the inclusion criteria were: (1) students aged 12 to 17 years and (2) students with complete responses for variables used in the analyses. After excluding those who did not meet the inclusion criteria, 122,284 student responses were included in the final analyses. The sample flow is presented in Fig. 1.

Note * Systematic reasons refer to the inability to administer the survey on the scheduled date due to the overburden of tasks on the trained teachers and the inability to use the computer rooms.

### Measures

#### Adherence to 24-hour movement guidelines

 The exposures were adherence to individual or different combinations of recommendations within 24-Hour Movement Guidelines [8], categorized into eight groups for weekdays and weekends: meeting none of the recommendations, meeting one individual recommendation (PA, SB, or sleep), meeting two recommendations (PA and SB, PA and sleep, or SB and sleep), and meeting all three recommendations (PA, SB, and sleep). Students self-reported their PA, SB, and sleep over the past seven days, and these were categorized as adhering or not adhering to the guidelines. SB and sleep were reported separately for weekdays and weekends, while PA was reported weekly and used for stratified analyses. The PA survey question asked: “In the past 7 days, on how many days did you do physical activities that increased your heart rate or made you breathe harder for a total of 60 minutes or more per day?” Responses ranged from 0 to 7 days, with those reporting MVPA for seven days considered adherent to the PA recommendation [8]. For SB, students reported hours and minutes spent sitting for recreational (e.g., watching TV, using the internet), with two hours or less considered meeting the SB recommendation [8]. Sleep duration was based on reported bedtimes and wake-up times (hours and minutes). Students aged 12 to 13 who slept 9 to 11 h, and those aged 14 to 17 who slept for 8 to 10 h, were considered as adhering to the sleep recommendation [8]. 

#### Anxiety

The psychological outcome examined was anxiety, assessed using the Generalized Anxiety Disorder-7 (GAD-7) scale [22]. The GAD-7 is a validated and reliable tool used to identify and assess symptoms of generalized anxiety disorder [22]. Due to its ease of administration, it has been used extensively in both clinical practice and research settings. The GAD-7 consists of seven items rated on a 4-point Likert scale ranging from 0 (*not at all*) to 3 (*nearly every day*). The total score ranges from 0 to 21, with individuals scoring 10 or higher considered to have high anxiety. Based on the scores, students were categorized in a binary manner (*yes* or *no*). A cut-off score of 10 or greater has high sensitivity and specificity for identifying cases of anxiety [22]. 

#### Intersectional identity groups

Participant characteristics used to group adolescents by intersectional identities, including biological sex (male, female), academic performance (i.e., “How would you describe your academic performance over the past 12 months?”), and family economic status (i.e., “How would you describe your family’s economic status?”) were self-reported by students. These variables were selected because they have been identified as important independent and jointly associated correlates of 24-hour movement behaviors in previous work [17]. Students rated their academic performance and family economic status on a 5-point Likert scale (low to high), which was then grouped into three categories: low (combining low and low-middle), middle, and high (combining middle-high and high). Intersectional groups were formed by combining sex (2 levels) with either academic performance (3 levels) or family economic status (3 levels).

#### Covariates

Age and body mass index (BMI) were self-reported to describe the sample characteristics and included as covariates. BMI (weight/height [kg/m²]) was calculated using self-reported height (cm) and weight (kg). Academic performance was included as a covariate when family economic status was considered an intersectional identity group, and vice versa.

### Statistical analyses

Descriptive statistics were used to describe the sample characteristics and the prevalence of exposures and outcomes, stratified by sex. To examine our hypotheses, logistic regression analyses were conducted to estimate weighted odds ratios (OR) and 95% confidence intervals (CI) for the association between meeting 24-Hour Movement Guidelines and anxiety across intersectional groups. From an epidemiological perspective, logistic regression is particularly beneficial for evaluating exposure-outcome relationships. It effectively accounts for multiple predictors, confounders, complex sampling designs, and weighting, which are often essential when analyzing survey-based or population-level data. Prior to the main analyses, multicollinearity was assessed using Variance Inflation Factor (VIF) values to ensure that no issues were present. To minimize bias concerns, make our findings more representative, and align with the underlying logistic regression assumptions, this study utilized a complex sampling design in SPSS to account for stratification, clustering, and weighting as suggested by the Korea CDC.

Using an intersectional approach, stratified analyses were performed through multiple logistic regression models to explore these associations. To ensure the adequacy of the sample size for each intersectional stratum, the Events Per Variable (EPV) criterion was applied, confirming that all strata met or exceeded the recommended threshold of 10 EPV [23]. Additionally, the associations were analyzed separately for weekdays and weekends due to well-known disparities in movement behaviors throughout the week [17, 24, 25]. To control for potential confounding effects, all covariates were included in all main analyses. An OR greater than 1 indicates higher odds of anxiety, while an OR less than 1 indicates lower odds. The significance level was set at *p* < 0.05. All analyses were performed using Statistical Package for the Social Sciences (SPSS; Version 29, Armonk, NY).

## Results

### Sample characteristics and proportions of exposure and outcome

The multicollinearity diagnostics indicated no violations. Additionally, EPV calculations for all intersectional strata exceeded the recommended threshold of 10 (lowest EPV 80), indicating that the sample size was sufficient for reliable estimates. Table 1 summarizes the sample characteristics and distributions for 122,284 participants (48.3% females). The mean age was similar for both sexes at around 14.8 years (SD = 1.5). Male students had a higher mean BMI (22.2, SD = 4.0) than their female counterparts (20.5, SD = 3.2). Academic performance was fairly even across sexes, with 40.0% of males and 38.5% of females categorized in the high-performance category. Most students were from middle- or high-income families, while 10.5% of male students and 10.2% of female students were from low-income families. Anxiety prevalence was higher in females (14.4%) than males (8.4%).

Weekday adherence to 24-Hour Movement Guidelines varied across the sample, with 49.7% of participants not adhering to any recommendations. Adherence rates were generally low: 2.8% for PA, 35.4% for SB, and 5.1% for sleep. Combined adherence rates were also low, with 2.5% adhering to both PA and SB, 0.4% adhering to both PA and sleep, and 3.6% adhering to both SB and sleep. Only 0.5% adhered to all three guidelines. Weekend adherence rates were slightly better, with 40.3% not adhering to any recommendations. Adherence to individual recommendations varied: 2.6% for PA, 11.3% for SB, and 34.0% for sleep. Combined adherence remained low, with 0.9% adhering to both PA and SB, 2.0% adhering to both PA and sleep, and 8.3% adhering to both SB and sleep. Adherence to all three recommendations was slightly higher on weekdays but still low at 0.7%. Sample sizes for all intersectional groups are provided in Supplementary file A.

**Table 1:** Participant characteristics

Characteristics	Males	Females	Total
Sex	63,063 (51.7%)	59,221 (48.3%)	-
Age	14.8 (1.5)	14.8 (1.5)	14.8 (1.5)
BMI	22.2 (4.0)	20.5 (3.2)	21.4 (3.7)
*Academic performance*			
Low	19,480 (30.9%)	17,968 (30.1%)	37,448 (30.5%)
Middle	18,461 (29.1%)	18,420 (31.4%)	36,881 (30.2%)
High	25,122 (40.0%)	22,833 (38.5%)	47,955 (39.3%)
*Family economic status*			
Low	6863 (10.5%)	6404 (10.2%)	13,267 (10.3%)
Middle	28,744 (44.9%)	29,790 (49.7%)	58,534 (47.2%)
High	27,456 (44.6%)	23,027(40.1%)	50,483 (42.4%)
*Adherence to guidelines (weekday)*			
None	28,966 (46.3%)	31,559 (53.4%)	60,525 (49.7%)
PA	2635 (4.1%)	909 (1.4%)	3544 (2.8%)
SB	20,699 (33.5%)	21,904 (37.5%)	42,603 (35.4%)
Sleep	4289 (6.4%)	2349 (3.7%)	6638 (5.1%)
PA + SB	2320 (3.6%)	775 (1.3%)	3095 (2.5%)
PA + Sleep	456 (0.6%)	81 (0.1%)	537 (0.4%)
SB + Sleep	3139 (4.7%)	1567 (2.4%)	4706 (3.6%)
All	559 (0.8%)	77 (0.1%)	636 (0.5%)
*Adherence to guidelines (weekend)*			
None	24,662 (39.0%)	24,522 (41.7%)	49,184 (40.3%)
PA	2548 (3.8%)	843 (1.3%)	3391 (2.6%)
SB	5842 (9.5%)	7671 (13.1%)	13,513 (11.3%)
Sleep	22,048 (35.0%)	19,574 (32.8%)	41,622 (34.0%)
PA + SB	779 (1.2%)	295 (0.5%)	1074 (0.9%)
PA + Sleep	2032 (3.1%)	516 (0.8%)	2548 (2.0%)
SB + Sleep	4541 (7.3%)	5612 (9.4%)	10,153 (8.3%)
All	611 (1.0%)	188 (0.3%)	799 (0.7%)
*Anxiety*			
No	57,876 (91.6%)	50,646 (85.6%)	108,522 (88.7%)
Yes	5187 (8.4%)	8575 (14.4%)	13,762 (11.3%)

Note: Values are presented as mean (SD) or N (%). N indicates unweighted sample counts, and % is weighted to the adolescent population

*BMI* Body mass index, *PA* Physical activity, *SB* Sedentary behavior

### Associations between adherence to 24-hour movement guidelines and anxiety across sex and academic performance

Table 2 presents the associations between weekday adherence to the 24-Hour Movement Guidelines and anxiety across intersectional identities, specifically at the intersection between sex and academic performance. Adherence to the sleep recommendation was associated with lower odds of reporting anxiety across all groups, with the strongest association found in males with high academic performance (OR: 0.50, 95%CI: 0.38–0.66) and females with high academic performance (OR: 0.48, 95%CI: 0.36–0.64). Female adolescents also exhibited a strong inverse relationship between sleep adherence and anxiety in middle academic performance group (OR: 0.91, 95%CI: 0.26–0.57). Combined adherence to SB and sleep recommendations was consistently associated with lower odds of reporting anxiety across all groups. However, adherence to the PA recommendation was linked to higher odds of reporting anxiety in females in low, middle, and high academic performance groups. Adhering to all recommendations was associated with lower odds of reporting anxiety only in low-performing males (OR: 0.47, 95%CI: 0.26–0.87), although a trend toward lower anxiety was seen in all groups.

**Table 2:** Associations between weekday adherence to 24-Hour movement guidelines and anxiety across sex and academic performance

	Male			Female		
	Low	Middle	High	Low	Middle	High
	OR (95% CI)	OR (95% CI)	OR (95% CI)	OR (95% CI)	OR (95% CI)	OR (95% CI)
Adherence to guidelines						
None	1.00 (reference)	1.00 (reference)	1.00 (reference)	1.00 (reference)	1.00 (reference)	1.00 (reference)
PA	1.10 (0.88–1.36)	1.16 (0.89–1.52)	1.20 (0.94–1.55)	1.62 (1.28–2.05)**	1.48 (1.04–2.10)*	1.43 (1.05–1.95)*
SB	0.92 (0.83–1.04)	0.93 (0.82–1.07)	1.00 (0.90–1.11)	1.01 (0.93–1.11)	0.91 (0.83-1.00)*	0.95 (0.87–1.04)
Sleep	0.81 (0.67–0.99)*	0.76 (0.58-1.00)*	0.50 (0.38–0.66)**	0.71 (0.58–0.87)**	0.57 (0.43–0.75)**	0.48 (0.36–0.64)**
PA + SB	0.96 (0.76–1.23)	1.19 (0.87–1.63)	0.95 (0.73–1.23)	1.48 (1.09–2.02)*	1.16 (0.77–1.74)	1.46 (1.08–1.96)*
PA + Sleep	0.55 (0.30-1.00)	0.51 (0.21–1.22)	0.54 (0.24–1.19)	0.91 (0.37–2.21)	1.32 (0.45–3.88)	0.18 (0.03–1.22)
SB + Sleep	0.67 (0.50–0.89)**	0.51 (0.36–0.71)**	0.63 (0.49–0.81)**	0.53 (0.39–0.73)**	0.39 (0.26–0.57)**	0.57 (0.43–0.77)**
All	0.47 (0.26–0.87)*	0.46 (0.19–1.11)	0.85 (0.42–1.69)	0.64 (0.21–1.96)	0.49 (0.07–3.31)	0.65 (0.18–2.28)

Note: Values are presented in OR (95% CI)

*PA* Physical activity, *SB* Sedentary behavior

* < 0.05, ** <0.01

Table 3 demonstrates that adherence to sleep guidelines during the weekend was consistently associated with lower odds of anxiety across all academic performance groups for both males and females. For males, significantly lower odds of reporting anxiety were observed in the low (OR: 0.73, 95%CI: 0.65–0.81), middle (OR: 0.83, 95%CI: 0.73–0.95), and high (OR: 0.89, 95%CI: 0.80–1.00) academic performance groups. Similarly, among females, lower odds of reporting anxiety were observed in the low (OR: 0.81, 95%CI: 0.74–0.88), middle (OR: 0.76, 95%CI: 0.69–0.84), and high (OR: 0.73, 95%CI: 0.67–0.80) academic performance groups. No statistically significant associations were found for adherence to SB recommendation alone. However, adhering to a combination of SB and sleep recommendations was associated with lower odds of reporting anxiety among males from low (OR: 0.65, 95%CI: 0.52–0.83) and middle (OR: 0.72, 95%CI: 0.56–0.92) academic performance groups, and in females across all academic performance groups: low (OR: 0.74, 95%CI: 0.64–0.87), middle (OR: 0.68, 95%CI: 0.57–0.81), and high (OR: 0.69, 95%CI: 0.59–0.80). Adherence to all three recommendations within the movement guidelines was associated with lower odds of reporting anxiety in males with low academic performance (OR: 0.58, 95%CI: 0.34–0.96). In contrast, adherence to PA recommendation was linked to higher odds of reporting anxiety, particularly in males from the middle academic performance group (OR: 1.31, 95%CI: 1.01–1.72) females from the low (OR: 1.67, 95%CI: 1.30–2.15), middle (OR: 1.49, 95%CI: 1.06–2.09), and high (OR: 1.44, 95%CI: 1.06–1.95) academic performance groups. However, adherence to both PA and sleep recommendation was associated with lower odds of reporting anxiety odds in males with the low academic performance only (OR: 0.73, 95%CI: 0.56–0.95).

**Table 3:** Associations between weekend adherence to 24-Hour movement guidelines and anxiety across sex and academic performance

	Male			Female		
	Low	Middle	High^a^	Low	Middle	High
	OR (95% CI)	OR (95% CI)	OR (95% CI)	OR (95% CI)	OR (95% CI)	OR (95% CI)
Adherence to guidelines						
None	1.00 (reference)	1.00 (reference)	1.00 (reference)	1.00 (reference)	1.00 (reference)	1.00 (reference)
PA	0.99 (0.79–1.24)	1.31 (1.01–1.72)*	1.16 (0.89–1.51)	1.67 (1.30–2.15)**	1.49 (1.06–2.09)*	1.44 (1.06–1.95)*
SB	1.02 (0.86–1.22)	0.91 (0.73–1.13)	1.03 (0.87–1.21)	0.95 (0.85–1.07)	1.04 (0.91–1.20)	0.94 (0.83–1.06)
Sleep	0.73 (0.65–0.81)**	0.83 (0.73–0.95)**	0.89 (0.80-1.00)*	0.81 (0.74–0.88)**	0.76 (0.69–0.84)**	0.73 (0.67–0.80)**
PA + SB	1.11 (0.76–1.61)	0.91 (0.54–1.55)	1.18 (0.76–1.85)	1.36 (0.85–2.17)	1.77 (0.95–3.30)	1.47 (0.93–2.33)
PA + Sleep	0.73 (0.56–0.95)*	0.75 (0.52–1.08)	0.88 (0.66–1.17)	1.00 (0.70–1.43)	0.81 (0.46–1.41)	0.93 (0.61–1.42)
SB + Sleep	0.65 (0.52–0.83)**	0.72 (0.56–0.92)**	0.94 (0.77–1.15)	0.74 (0.64–0.87)**	0.68 (0.57–0.81)**	0.69 (0.59–0.80)**
All	0.58 (0.34–0.96)*	0.88 (0.44–1.79)	0.87 (0.49–1.51)	1.22 (0.71–2.12)	0.67 (0.27–1.66)	0.97 (0.48–1.99)

Note: Values are presented in OR (95% CI)

*PA* Physical activity, *SB* Sedentary behavior

^a^These values should be interpreted cautiously due to the insignificant overall effect

* < 0.05, ** <0.01

### Associations between adherence to 24-hour movement guidelines and anxiety across sex and family economic status intersectional groups

Table 4 illustrates the associations between weekday adherence to 24-Hour Movement Guidelines and anxiety among adolescents, stratified by sex and family economic status. Adherence to the sleep recommendation was consistently associated with lower odds of reporting anxiety across all economic statuses for both sexes. Specifically, lower odds of reporting anxiety were observed in males from middle (OR: 0.69, 95%CI: 0.57–0.84) and high (OR: 0.64, 95%CI: 0.51–0.80) economic status groups, and in females, particularly those from high economic status (OR: 0.48, 95%CI: 0.37–0.63). Adhering to the sleep and SB recommendation was also associated with lower odds of reporting anxiety among males from low (OR: 0.59, 95%CI: 0.38–0.91) and middle (OR: 0.55, 95%CI: 0.42–0.72) economic statuses, and among females from high economic status only (OR: 0.46, 95%CI: 0.34–0.64). Conversely, adherence to the PA recommendation was associated with higher odds of reporting anxiety across all intersectional groups, particularly among females from middle (OR: 1.68, 95%CI: 1.33–2.12) and high (OR: 1.43, 95%CI: 1.09–1.87) economic statuses. However, adhering to the PA and sleep recommendations was associated with lower odds of reporting anxiety among males from middle (OR: 0.32, 95%CI: 0.16–0.66) and high (OR: 0.34, 95%CI: 0.14–0.85) economic statuses. Adherence to PA and sleep recommendations was associated with higher odds of reporting anxiety among males with low economic status. While adherence to all recommendations was associated with lower odds of reporting anxiety, these associations were not statistically significant.

**Table 4:** Associations between weekday adherence to 24-Hour movement guidelines and anxiety across sex and economic status

	Male			Female		
	Low	Middle	High	Low^a^	Middle	High
	OR (95% CI)	OR (95% CI)	OR (95% CI)	OR (95% CI)	OR (95% CI)	OR (95% CI)
Adherence to guidelines						
None	1.00 (reference)	1.00 (reference)	1.00 (reference)	1.00 (reference)	1.00 (reference)	1.00 (reference)
PA	1.17 (0.86–1.60)	1.15 (0.92–1.44)	1.10 (0.89–1.36)	1.29 (0.87–1.92)	1.68 (1.33–2.12)**	1.43 (1.09–1.87)*
SB	0.10 (0.85–1.17)	0.96 (0.87–1.06)	0.94 (0.85–1.05)	1.09 (0.96–1.24)	0.95 (0.88–1.02)	0.92 (0.85-1.00)
Sleep	0.88 (0.64–1.21)	0.69 (0.57–0.84)**	0.64 (0.51–0.80)**	0.57 (0.42–0.79)**	0.70 (0.56–0.86)**	0.48 (0.37–0.63)**
PA + SB	1.03 (0.72–1.47)	1.01 (0.79–1.29)	0.97 (0.77–1.23)	1.33 (0.85–2.08)	1.50 (1.11–2.01)**	1.26 (0.94–1.69)
PA + Sleep	2.22 (1.07–4.62)*	0.32 (0.16–0.66)**	0.34 (0.14–0.85)*	2.09 (0.66–6.59)	0.65 (0.23–1.88)	0.49 (0.15–1.67)
SB + Sleep	0.59 (0.38–0.91)*	0.55 (0.42–0.72)**	0.67 (0.53–0.86)**	0.58 (0.38–0.90)*	0.50 (0.38–0.66)**	0.46 (0.34–0.64)**
All	0.41 (0.14–1.19)	0.47 (0.25–0.90)*	0.72 (0.40–1.30)	-	0.63 (0.20–1.95)	0.72 (0.27–1.90)

Note: Values are presented in OR (95% CI)

*PA* Physical activity, *SB* Sedentary behavior

^a^Among females with low family economic status, there were very few observations, and the model provided an extreme estimate, so we did not report the values

* < 0.05, ** <0.01

Table 5 demonstrates the findings from weekend days stratified by sex and family economic status. Adherence to the sleep recommendation was associated with lower odds of reporting anxiety across all economic status groups, with stronger association observed in females from high economic status families (OR: 0.72, 95%CI: 0.66–0.79). Males also showed significant reductions in anxiety across low, middle, and high economic status groups (OR: 0.84, 95%CI: 0.71–0.99; OR: 0.81, 95%CI: 0.73–0.89; OR: 0.81, 95%CI: 0.73–0.91, respectively). Adhering to both SB and sleep recommendations was also associated with lower odds of reporting anxiety in both sexes, with the strongest association observed among females from high economic status families (OR: 0.63, 95%CI: 0.54–0.73). Conversely, adherence to the PA recommendation was associated with higher odds of reporting anxiety in females, regardless of family economic status.

**Table 5:** Associations between weekend adherence to 24-Hour movement guidelines and anxiety across sex and economic status

	Male			Female		
	Low^a^	Middle	High	Low	Middle	High
	OR (95% CI)	OR (95% CI)	OR (95% CI)	OR (95% CI)	OR (95% CI)	OR (95% CI)
Adherence to guidelines						
None	1.00 (reference)	1.00 (reference)	1.00 (reference)	1.00 (reference)	1.00 (reference)	1.00 (reference)
PA	1.19 (0.87–1.63)	0.97 (0.76–1.25)	1.20 (0.96–1.49)	1.82 (1.23–2.70)**	1.52 (1.18–1.96)**	1.42 (1.07–1.88)*
SB	0.94 (0.73–1.22)	1.10 (0.94–1.30)	0.91 (0.78–1.07)	1.12 (0.93–1.35)	0.96 (0.86–1.07)	0.93 (0.83–1.05)
Sleep	0.84 (0.71–0.99)*	0.81 (0.73–0.89)**	0.81 (0.73–0.91)**	0.86 (0.75–0.98)*	0.78 (0.73–0.84)**	0.72 (0.66–0.79)**
PA + SB	1.09 (0.59–2.02)	1.48 (1.03–2.13)*	0.79 (0.51–1.22)	1.61 (0.79–3.28)	1.69 (1.09–2.60)*	1.24 (0.79–1.95)
PA + Sleep	0.80 (0.52–1.23)	0.79 (0.61–1.02)	0.76 (0.59–1.02)	0.60 (0.32–1.13)	1.12 (0.78–1.59)	0.87 (0.59–1.29)
SB + Sleep	0.69 (0.50–0.95)*	0.77 (0.64–0.92)**	0.82 (0.67-1.00)*	0.79 (0.63-1.00)*	0.73 (0.64–0.83)**	0.63 (0.54–0.73)**
All	1.08 (0.54–2.15)	0.72 (0.42–1.24)	0.63 (0.37–1.09)	0.70 (0.27–1.81)	1.32 (0.71–2.45)	0.95 (0.52–1.75)

Note: values are presented in OR (95% CI)

*PA* Physical activity, *SB* Sedentary behavior

^a^These values should be interpreted cautiously due to the insignificant overall effect

* < 0.05, ** <0.01

## Discussion

Associations between 24-hour movement behaviors (i.e., PA, SB, and sleep) and generalized anxiety have been studied previously [16], but the specific relationships across intersectional strata among adolescents remain underexplored. This cross-sectional study, utilizing secondary analysis of a representative adolescent sample from Korea during the COVID-19 pandemic, examined the associations between adherence to 24-Hour Movement Guidelines and anxiety across intersectional strata, including sex, academic performance, and family economic status. To the best of our knowledge, this study is among the first to employ an intersectional framework to explore the varied associations between movement behaviors and anxiety across intersectional identities among adolescents. Our findings partially supported the initial hypotheses. While adherence to some recommendations was associated with reduced anxiety compared to non-adherence, following all three recommendations, or a combination of two, showed potential protective associations. Furthermore, although these protective associations were not consistent across all intersectional groups, adherence to two movement recommendations demonstrated stronger associations with reduced anxiety in certain intersectional strata compared to adherence to just one.

Our findings highlight the potential protective role of obtaining adequate sleep, both on weekdays and weekends, in anxiety across all intersectional groups. Combined adherence to sleep and SB recommendations was consistently associated with lower anxiety. While adherence to the SB recommendation alone showed inconsistent results, the combination of sleep and SB adherence had a stronger association with reduced anxiety than adhering to either recommendation individually. These results align with previous study finding [16] and underscore the crucial role of adequate sleep in safeguarding adolescent mental health, with its benefits being further enhanced when coupled with adherence to the SB recommendation.

Within the 24-hour movement paradigm asserting that movement behaviors influence one another [26], our findings may indicate that adolescents who did not achieve adequate sleep may engage in more recreational sedentary screen time [27], increasing their levels of anxiety. During the COVID-19 pandemic, public health restrictions limited in-person social interactions, likely prompting adolescents to use screen-based devices for virtual interactions, further increasing the use of sedentary screen time. Moreover, studies have shown that social media use is associated with heightened anxiety and other mental health conditions [28]. Therefore, it is crucial to advise adolescents not to trade off sleep for prolonged SB (e.g., screen time), as sufficient sleep is key to positive mental health. For instance, a study using a representative U.S. adolescent sample found that insufficient sleep duration (less than 7 h) was associated with mental disorders [29]. Another study presented that replacing sedentary/screen time and MVPA with sleep improved mental health, particularly among adolescents not meeting sleep guidelines [30]. Additionally, it is essential to equip adolescents with strong digital literacy skills, enabling them to navigate social media safely and avoid exposure to online bullying, distressing news, and harmful content.

This study found that male and female adolescents with high academic performance and family economic status were less likely to experience anxiety when they achieved adequate sleep during weekdays, particularly compared to their peers with low academic performance and family economic status. This finding echoes earlier studies that identified links between low social positions and poor sleep, as well as between poor sleep and adverse health outcomes [31]. Several environmental factors, such as household and neighborhood characteristics, may contribute to this observation and are well documented in the literature as key determinants of healthy sleep [32]. For example, adolescents from higher socioeconomic groups are more likely to benefit from home environments conducive to healthier sleep, such as quiet, safe surroundings and less stress related to financial stability and career prospects, which lowers exposure to risky behaviors. Additionally, neighborhood factors like physical (e.g., population density, air quality, noise levels) and social environment (e.g., safety, social cohesion) play pivotal roles in supporting or hindering healthy sleep and overall well-being [32]. Therefore, sociodemographic disadvantages are likely associated with poorer sleep quality [32–34], which may explain the varying protective associations against anxiety across different social groups.

On weekends, both sexes exhibited similar protective patterns of sleep adherence, with some additional unique observations also emerging. On weekends, boys with low academic performance and family economic status experienced less anxiety when they adhered to the sleep recommendation alone or in combination with the SB recommendation, compared to their counterparts from higher status groups. This result contrasts with those of prior studies [31], possibly because Korea’s highly competitive educational environment imposes unique stressors on adolescents from higher socioeconomic groups. In 2023, 78.5% of Korean adolescents participated in extracurricular activities, predominantly in math, English, and Korean literature, spending an average of 7.3 h per week [35]. This intense academic pressure may contribute to higher anxiety levels in more advantaged groups, as participation in private education is closely related to household economic status, requiring substantial time and financial investment [36]. Therefore, these trends may impact weekend sleep and anxiety symptoms among higher-income students, who face accumulated homework and increased extracurricular demands over the weekend, leading to heightened anxiety in high economic status and academic performance groups. These findings suggest that the benefits of modifiable behaviors, such as obtaining adequate sleep and limiting recreational SB like screen time, are generally associated with lower anxiety, regardless of social gradients. Future studies should consider incorporating measures of sleep quality, such as sleep disturbances, to provide a more comprehensive understanding of the relationship between sleep metrics and anxiety.

In terms of PA, our findings indicated that adhering to the PA recommendation alone was not linked to lower anxiety for any intersectional identity groups on either weekdays or weekends. This PA-anxiety relationship is unexpected, as previous studies have consistently shown a positive association between meeting PA guidelines and improved mental health outcomes [16, 37]. While PA adherence alone was not shown to safeguard against anxiety, reduced odds of anxiety were observed when adolescents adhered to both PA and sleep recommendations. The negative association between the PA adherence and anxiety can be understood in light of previous research suggesting that the benefits of PA may be amplified when other healthy habits also exist such as getting sufficient sleep [30, 37]. This implies that the positive association between PA and anxiety may be strengthened with adequate sleep among Korean adolescents. For anxious adolescents, strictly following the PA recommendation only may not always be beneficial. However, even light-intensity PA like yoga can alleviate anxiety symptoms [38, 39], and any amount of exercise is better than none [38], especially with growing evidence supporting the positive effects of mindfulness-based exercises on mental health.

Interestingly, increased odds of anxiety in low-income households of both sexes were still noted when adolescents adhered to PA and sleep recommendations during weekdays. Several factors may contribute to this finding. For example, factors such as poorer sleep quality may further heighten anxiety levels in these groups [31]. Additionally, adolescents from low-income households often encounter extra stressors (e.g., unstable living conditions and food insecurity), and may lack family social support, which can increase anxiety regardless of healthy behaviors [40], which can elevate anxiety regardless of healthy behaviors. Thus, while meeting PA and sleep recommendations is generally more health-promoting compared to adhering to PA alone among Korean adolescents, the broader socio-environmental context, especially in lower-income settings, may undermine or mask these benefits, resulting in continued high anxiety levels. Additionally, our findings should be considered within the context of the COVID-19 pandemic, during which worsening economic conditions and deteriorating home environments were more prevalent among low-income households, acting as additional stressors [41]. The pandemic also significantly disrupted daily routines and may have impacted movement behaviors among Korean adolescents. The reduction in social interactions due to social distancing, school closures, and mask mandates may have made adolescents less comfortable participating in PA, particularly due to breathing discomfort caused by masks and a lack of social support. These factors could have contributed to heightened anxiety levels during this period [42, 43]. 

We observed higher odds of anxiety among female adolescents compared to males when adhering to PA guidelines, despite of their academic performance or family economic status. These findings align with previous study that examined the bidirectional longitudinal association between PA and mental health, which showed that higher MVPA was linked to greater anxiety in females, whereas males exhibited lower levels of anxiety [44]. These observations underscore the complex relationship between socioeconomic factors, health behaviors, and mental health outcomes, highlighting the importance of considering these variables in research and interventions aimed at reducing adolescent anxiety. To address this complexity, researchers, educators, and health professionals should account for both the mental health status and socioeconomic backgrounds of adolescents when recommending MVPA. This approach ensures that interventions are tailored to meet the diverse needs of adolescents, promoting better mental health outcomes across various sociodemographic groups.

This study shed light on the practical implications of understanding the complexity of associations between 24-hour movement behaviors and anxiety, particularly across intersectional identities. Our observations emphasize the importance of considering factors such as sex, economic status, and academic performance in understanding the dynamics of anxiety among adolescents. This study presents several notable strengths. First, the use of an intersectional framework enabled a comprehensive examination of how multiple identity factors interact to influence anxiety outcomes. This approach allowed us to identify specific at-risk subpopulations, paving the way for more targeted and effective interventions. Second, the use of a representative, population-based sample of Korean adolescents ensures generalizability and external validity of the findings. Third, the complex data design analysis provides more accurate and representative insights into the relationships between adherence to 24-Hour Movement Guidelines and anxiety among adolescents.

However, several limitations should be considered when interpreting the findings. First, the cross-sectional design prevents determining temporal relationships, limiting our ability to infer causality. Second, as a survey-based study, there is potential for recall bias, which may affect the accuracy of the self-reported data. Third, unmeasured confounding factors could influence the observed associations between adherence to 24-Hour Movement Guidelines and anxiety among adolescents. Additionally, while the KYRBS excluded students with prolonged absences, special needs, or literacy challenges to ensure the accuracy and reliability of self-reported data and facilitate comparisons with international health data, it should be noted that the results may not fully represent Korean adolescents with diverse backgrounds and experiences. Despite these limitations, the use of representative sample and complex sample design mitigates bias concerns in this study. Lastly, because this study used data collected during the pandemic (2020–2022), the findings only reflect behaviors and perceptions specific to that period; therefore, caution is needed when generalizing these results to post-pandemic or non-pandemic contexts. Finally, while the 24-Hour Movement Behavior Guidelines are an evidence-based approach, using standardized thresholds (e.g., 60 min of daily MVPA) may not fully capture the complexities of movement behavior. However, it should be noted that the 24-Hour Movement Guidelines provide significant practical benefits and are consistent with widely accepted public health guidelines. Utilizing these well-known cutoffs enables easy comparison of our findings with other studies using the same guidelines.

Future observational studies should use prospective data from prospective cohort designs to establish temporal relationships between modifiable movement behaviors and anxiety. In future research, it is crucial to use objectively measured movement behaviors, such as accelerometers or fitness wearables, to accurately assess PA dose in terms of duration, frequency, and intensity. Although the stratified analysis in this study identified important patterns, it may not fully capture the complexities arising from multiple intersecting identity factors. To address this, future research should incorporate advanced intersectional methodologies to deepen our understanding of these dynamics and enable the development of targeted interventions for at-risk youth. Additionally, high-quality randomized controlled trials are needed to enhance our understanding of the benefits of different modes and levels of PA for anxiety among adolescents, given the limited evidence available. Furthermore, because Korea is a relatively homogeneous society, data on race and ethnicity were not available for this study. However, as Korea becomes increasingly diverse, we encourage future researchers to include measures of multiple identities (e.g., race/ethnicity, gender identity) to provide a more holistic understanding of adolescent mental health disparities in diverse settings. Additionally, childhood experiences, such as interactions with family or friends and the presence of social support, are crucial determinants of health. These factors can help clarify the pathways that influence the relationship between movement behaviors and mental health. Consequently, we recommend that future research also explore these aspects.

## Conclusion

Generalized anxiety may be lessened when the sleep recommendation is met with SB recommendation. Using a large, representative data and employing an intersectional lens, our study explored the associations between adhering to 24-Hour Movement Guidelines and anxiety among South Korean adolescents across different social strata. The findings offer valuable insights into preventive non-pharmacological measures that could inform mental health promotion strategies. To translate these findings into practice, health practitioners, policymakers, and educators should educate adolescents about the numerous health benefits associated with the 24-Hour Movement Guidelines, as well as prioritize interventions that promote adequate, high-quality sleep and reduce SB among adolescents. For instance, school-based programs emphasizing sleep hygiene, screen time management, and PA tailored to adolescents’ diverse needs can be implemented. Additionally, recognizing that the protective effects of adhering to 24-hour movement behaviors may vary across social groups can help inform targeted strategies to address the unique needs of these groups and reduce health disparities. Sex-specific interventions may also be necessary to address the higher anxiety observed in Korean female adolescents adhering to PA guidelines. Moreover, tailoring these interventions to address the unique challenges faced by specific intersectional groups (e.g., low socioeconomic status or high academic pressures) can enhance their effectiveness and contribute to reducing mental health disparities.

## Supplementary Information

Supplementary Material 1. File name: Supplementary File A. File format: PDF. Title of data: Sample Size of Intersectional Groups. Description of data: This file presents tables displaying the sample sizes of intersectional groups categorized by adherence to the 24-Hour Movement Guidelines (24HMB)

## Data Availability

Data used in this study is publicly available online at the Korea Disease Control and Prevention Agency.

## Abbreviations

PA: Physical activity
SB: Sedentary behavior
MVPA: Moderate-to-vigorous physical activity
CDC: Centers for Disease Control and Prevention
