# Supplementary material for: Associations between adhering to 24-hour movement guidelines and anxiety among adolescents across intersectional identities: KYRBS 2020–2022
Source: BMC Public Health. 2025 Sep 24;25:3103. doi: 10.1186/s12889-025-24215-9 (PMC12462027; doi:10.1186/s12889-025-24215-9)
Supplement: Supplementary file 1 — Supplementary Material 1. File name: Supplementary File A. File format: PDF. Title of data: Sample Size of Intersectional Groups. Description of data: This file presents tables displaying the sample sizes of intersectional groups categorized by adherence to the 24-Hour Movement Guidelines (24HMB) [file 12889_2025_24215_MOESM1_ESM.docx]

Manuscript Title: Associations between Adhering to 24-Hour Movement Guidelines and Anxiety Among Adolescents Across Intersectional Identities: KYRBS 2020-2022

File name: Supplementary File A

File format: PDF

Title of data: Sample Size of Intersectional Groups

Description of data: This file presents tables displaying the sample sizes of intersectional groups categorized by adherence to the 24-Hour Movement Guidelines (24HMB).

Table S1. Sample sizes of intersectional groups by sex, income, and 24-hour movement behavior (weekday)

|  | Male | | | Female | | |
| --- | --- | --- | --- | --- | --- | --- |
|  | Low | Middle | High | Low | Middle | High |
| Adherence to guidelines |  |  |  |  |  |  |
| None | 3452  (51.1) | 13838  (48.7) | 11676  (42.9) | 3478  (54.8) | 16267  (54.9) | 11814  (51.2) |
| PA | 288  (4.1) | 1098  (3.7) | 1249  (4.4) | 128  (1.9) | 402  (1.2) | 379  (1.6) |
| SB | 2073  (30.0) | 9048  (31.9) | 9578  (35.8) | 2208  (35.0) | 10742  (36.4) | 8954  (39.6) |
| SL | 433  (6.2) | 2047  (6.6) | 1809  (6.2) | 277  (3.8) | 1231  (3.8) | 841  (3.4) |
| PA + SB | 244  (3.5) | 894  (3.1) | 1182  (4.1) | 109  (1.6) | 314  (1.0) | 352  (1.4) |
| PA + SL | 42  (0.5) | 212  (0.7) | 202  (0.7) | 14  (0.2) | 37  (0.1) | 30  (0.1) |
| SB + SL | 290  (4.0) | 1386  (4.6) | 1463  (4.9) | 185  (2.7) | 766  (2.3) | 616  (2.5) |
| All | 41  (0.6) | 221  (0.7) | 297  (1.0) | 5  (0.0) | 31  (0.1) | 41  (0.2) |

Note: Values are presented in unweighted counts and weighted percentages; PA = physical activity; SB = sedentary behavior; SL = sleep

Table S2. Sample sizes of intersectional groups by sex, income, and 24-hour movement behavior (weekend)

|  | Male | | | Female | | |
| --- | --- | --- | --- | --- | --- | --- |
|  | Low | Middle | High | Low | Middle | High |
| Adherence to guidelines |  |  |  |  |  |  |
| None | 2794  (40.5) | 11393  (39.6) | 10475  (38.2) | 2620  (40.7) | 12328  (41.7) | 9574  (42.1) |
| PA | 272  (4.0) | 1043  (3.5) | 1233  (4.2) | 119  (1.7) | 372  (1.2) | 352  (1.4) |
| SB | 605  (8.9) | 2408  (8.5) | 2829  (10.7) | 827  (13.1) | 3663  (12.4) | 3181  (14.1) |
| SL | 2282  (35.2) | 10543  (36.8) | 9123  (33.2) | 2062  (32.2) | 10188  (34.2) | 7324  (31.3) |
| PA + SB | 83  (1.2) | 276  (0.9) | 420  (1.5) | 39  (0.5) | 116  (0.4) | 140  (0.6) |
| PA + SL | 193  (2.6) | 875  (2.9) | 964  (3.4) | 70  (1.0) | 230  (0.7) | 216  (0.8) |
| SB + SL | 467  (6.6) | 1975  (6.9) | 2099  (7.8) | 639  (10.2) | 2827  (9.3) | 2146  (9.3) |
| All | 67  (0.9) | 231  (0.8) | 313  (1.1) | 28  (0.5) | 66  (0.2) | 94  (0.4) |

Note: Values are presented in unweighted counts and weighted percentages; PA = physical activity; SB = sedentary behavior; SL = sleep

Table S3. Sample sizes of intersectional groups by sex, academic, and 24-hour movement behavior (weekday)

|  | Male | | | Female | | |
| --- | --- | --- | --- | --- | --- | --- |
|  | Low | Middle | High | Low | Middle | High |
| Adherence to guidelines |  |  |  |  |  |  |
| None | 9937  (51.7) | 8688  (47.5) | 10361  (41.3) | 10369  (58.0) | 10047  (54.7) | 11143  (48.8) |
| PA | 994  (5.0) | 758  (4.0) | 883  (3.4) | 390  (2.0) | 233  (1.2) | 286  (1.2) |
| SB | 5201  (27.1) | 5874  (32.4) | 9624  (39.1) | 5593  (31.6) | 6666  (36.8) | 9645  (42.8) |
| SL | 1455  (7.1) | 1354  (6.8) | 1480  (5.5) | 852  (4.4) | 730  (3.6) | 767  (3.1) |
| PA + SB | 756  (3.7) | 603  (3.3) | 961  (3.7) | 258  (1.4) | 207  (1.0) | 310  (1.3) |
| PA + SL | 164  (0.7) | 123  (0.6) | 169  (0.6) | 38  (0.2) | 22  (0.1) | 21  (0.1) |
| SB + SL | 767  (3.7) | 929  (4.7) | 1443  (5.5) | 437  (2.2) | 498  (2.5) | 632  (2.6) |
| All | 206  (1.0) | 152  (0.8) | 201  (0.8) | 31  (0.2) | 17  (0.1) | 29  (0.1) |

Note: Values are presented in unweighted counts and weighted percentages; PA = physical activity; SB = sedentary behavior; SL = sleep

Table S4. Sample sizes of intersectional groups by sex, academic, and 24-hour movement behavior (weekend)

|  | Male | | | Female | | |
| --- | --- | --- | --- | --- | --- | --- |
|  | Low | Middle | High | Low | Middle | High |
| Adherence to guidelines |  |  |  |  |  |  |
| None | 7728  (39.6) | 7308  (39.4) | 2703  (38.3) | 7545  (42.1) | 7657  (41.9) | 9320  (41.3) |
| PA | 910  (4.4) | 724  (3.8) | 914  (3.4) | 324  (1.7) | 233  (1.2) | 286  (1.2) |
| SB | 1550  (8.0) | 1589  (8.8) | 9629  (11.2) | 2207  (12.3) | 2273  (12.5) | 3191  (14.3) |
| SL | 6866  (35.9) | 6591  (35.9) | 8591  (33.8) | 5834  (32.6) | 6262  (33.7) | 7478  (32.2) |
| PA + SB | 268  (1.3) | 217  (1.2) | 294  (1.2) | 102  (0.6) | 75  (0.4) | 118  (0.5) |
| PA + SL | 723  (3.5) | 544  (2.8) | 765  (2.9) | 206  (1.0) | 133  (0.7) | 177  (0.7) |
| SB + SL | 1216  (6.1) | 1337  (7.2) | 1988  (8.2) | 1665  (9.2) | 1749  (9.4) | 2198  (9.6) |
| All | 219  (1.2) | 151  (0.8) | 241  (0.9) | 85  (0.5) | 38  (0.2) | 65  (0.3) |

Note: Values are presented in unweighted counts and weighted percentages; PA = physical activity; SB = sedentary behavior; SL = sleep
